# Understanding Mechanisms of GLI-Mediated Transcription during Craniofacial Development and Disease Using the Ciliopathic Mutant, *talpid*^*2*^

**DOI:** 10.3389/fphys.2016.00468

**Published:** 2016-10-17

**Authors:** Ya-Ting Chang, Praneet Chaturvedi, Elizabeth N. Schock, Samantha A. Brugmann

**Affiliations:** ^1^Division of Plastic Surgery, Department of Surgery, Cincinnati Children's Hospital Medical CenterCincinnati, OH, USA; ^2^Division of Developmental Biology, Department of Pediatrics, Cincinnati Children's Hospital Medical CenterCincinnati, OH, USA

**Keywords:** primary cilia, craniofacial, *talpid*^*2*^, *c2cd3*, ciliopathies, GLI

## Abstract

The primary cilium is a ubiquitous, microtubule-based organelle that cells utilize to transduce molecular signals. Ciliopathies are a group of diseases that are caused by a disruption in the structure or function of the primary cilium. Over 30% of all ciliopathies are primarily defined by their craniofacial phenotypes, which typically include midfacial defects, cleft lip/palate, micrognathia, aglossia, and craniosynostosis. The frequency and severity of craniofacial phenotypes in ciliopathies emphasizes the importance of the cilium during development of the craniofacial complex. Molecularly, many ciliopathic mutants, including the avian *talpid*^*2*^ (*ta*^*2*^), report pathologically high levels of full-length GLI3 (GLI3_FL_), which can go on to function as an activator (GLI_A_), and reduced production of truncated GLI3 (GLI3_T_), which can go on to function as a repressor (GLI_R_). These observations suggest that the craniofacial phenotypes of ciliary mutants like *ta*^*2*^ are caused either by excessive activity of the GLI_A_ or reduced activity of GLI_R_. To decipher between these two scenarios, we examined GLI3 occupation at the regulatory regions of target genes and subsequent target gene expression. Using *in silico* strategies we identified consensus GLI binding regions (GBRs) in the avian genome and confirmed GLI3 binding to the regulatory regions of its targets by chromatin immunoprecipitation (ChIP). In *ta*^*2*^ mutants, there was a strikingly low number of GLI3 target genes that had significantly increased expression in facial prominences compared to the control embryo and GLI3 occupancy at GBRs associated with target genes was largely reduced. *In vitro* DNA binding assays, further supported ChIP results, indicated that the excessive GLI3_FL_ generated in *ta*^*2*^ mutants did not bind to GBRs. In light of these results, we explored the possibility of GLI co-regulator proteins playing a role in regulatory mechanism of GLI-mediated transcription. Taken together our studies suggest that craniofacial ciliopathic phenotypes are produced via reduced GLI_T_ production, allowing for target gene transcription to be mediated by the combinatorial code of GLI co-regulators.

## Introduction

Primary cilia are ubiquitous organelles that serve as cellular hubs for transduction of numerous signaling pathways. Most notably, cilia have been identified as transducers of the Hedgehog (Hh) pathway. Identification of the primary cilium as a signaling hub for the Hh pathway came from seminal experiments reporting that anterograde and retrograde intraflagellar transport (IFT) proteins in the cilium are required for Sonic Hedgehog (SHH) signal propagation (Huangfu et al., 2003; Huangfu and Anderson, 2005). When SHH ligand is present, it binds to its receptor Patched (PTCH), thus allowing Smoothened (SMO) to localize and accumulate in the primary cilium (Corbit et al., 2005; Rohatgi et al., 2007). Activated, ciliary SMO, in concert with Kif7, then promotes the dissociation of GLI from Suppressor of Fused (SUFU) (Humke et al., 2010; Tukachinsky et al., 2010; Li et al., 2012) and the subsequent post-translational processing of GLI proteins necessary for their function as activators and repressors (Goetz and Anderson, 2010).

In vertebrates, there are three members of the GLI transcription factor family: GLI1, GLI2, and GLI3. GLI1 and GLI2 are considered transcriptional activators, whereas GLI3 mostly behaves as a repressor. However, there have been examples of GLI2 functioning as a repressor in the absence of GLI3, and GLI3 functioning as an activator in the absence of GLI2 (Mo et al., 1997; Theil et al., 1999; Tole et al., 2000; Bai and Joyner, 2001; Persson et al., 2002; Rallu et al., 2002; Buttitta et al., 2003; Motoyama et al., 2003; Bai et al., 2004; Lei et al., 2004; McDermott et al., 2005; Pan et al., 2009). Full-length GLI2 and GLI3 can be processed via phosphorylation and other post-translational modifications into the activator isoform (GLI_A_) or truncated into the repressor isoform (GLI_R_) (Wang et al., 2000; Pan et al., 2006). Inhibition of GLI processing prevents production of GLI_A_ and GLI_R_ isoforms. Thus, an essential role of primary cilia is to establish the ratio of GLI_A_ to GLI_R_ proteins (Haycraft et al., 2005; Liu et al., 2005), which in turn controls transcription of SHH target genes.

Three basic models have been proposed to depict the potential mechanism of how SHH target genes are activated by a gradient of GLI isoforms: (1) the ratio sensing model, (2) the threshold repression model and (3) the threshold activation model (Falkenstein and Vokes, 2014). Ratio sensing, as the name implies, is based on the ratio of GLI_A_ and GLI_R_ rather than concentration of either. The net balance of GLI_A_ to GLI_R_ then determines if, and the extent to which, a target is activated or repressed. The other two models suggest threshold-specific mechanisms. The threshold activation model suggests that SHH targets are activated when GLI_A_ reaches a threshold-specific concentration. On the other hand, in the threshold repression model (de-repression), the activation of SHH target genes is dictated by the alleviation of repression via loss of GLI_R_.

The *talpid*^*2*^ (*ta*^*2*^) is a naturally occurring avian mutant that is best characterized by severe polydactyly and its oral-facial phenotype (Abbott et al., 1959, 1960; Dvorak and Fallon, 1991; Schneider et al., 1999). The face of affected *ta*^*2*^ embryos is characterized by a dysmorphic frontonasal prominence, facial clefting, hypoplastic maxillary prominences, incomplete fusion of the primary palate and hypoglossia (Chang et al., 2014). Our recent work determined that the *ta*^*2*^ mutation affected ciliogenesis via a 19 bp deletion in *C2CD3* (Brugmann et al., 2010; Chang et al., 2014), a centriolar protein required for ciliogenesis (Hoover et al., 2008). Our studies also determined that the *ta*^*2*^ mutant genetically, biochemically and phenotypically phenocopied the human craniofacial ciliopathy, Oral-facial-digital syndrome 14 (OFD14) (Schock et al., 2015). *ta*^*2*^ embryos, similar to many other ciliopathies, have a significant increase in GLI3_FL_ and a reduction in the amount GLI_T_ (Chang et al., 2014). However, the mechanism by which this disruption in GLI isoform production affects expression of GLI targets in the developing craniofacial complex remains unknown.

Herein, we utilize a combination of several biochemical techniques to determine the impact loss of cilia has on GLI function. Specifically, we examine the expression of GLI target genes and occupation of GLI binding regions (GBRs) associated with those targets in the developing frontonasal, maxillary and mandibular prominences (FNP, MXP, and MNP, respectively) in order to uncover the mechanism by which GLI mediated transcription is being impacted in *ta*^*2*^ mutants. Understanding the full extent of molecular disruptions in *ta*^*2*^ mutants will hopefully guide future therapeutic strategies for craniofacial ciliopathies, a rapidly growing group of disorders that currently have little to no therapeutic treatment.

## Materials and methods

### Embryo preparation

*talpid*^*2*^ (*ta*^*2*^) heterozygous carriers were mated, eggs were collected and shipped from the UC Davis Avian Sciences Department. Embryos were incubated at 37°C for approximately 5–7 days when embryos reached Hamburger Hamilton stage 25–31 (HH25-31).

### Quantitative RT-PCR of GLI targets

FNPs, MXPs, and MNPs were harvested from day 5 chick embryos. mRNA was prepared with TRIzol reagent (Thermo Fisher Scientific), and then converted to complementary DNA through reverse transcription reaction (High-Capacity cDNA Reverse Transcription Kit, Applied Biosystems™). Different amounts of cDNA (40, 20, 10 and 5 ng) was used for quantitative PCR to test PCR efficiency and a linear range of duplication (SsoAdvanced™ Universal SYBR® Green Supermix, BioRad). Expression of genes known to play a role in craniofacial development were examined with the following primer sets: *ALX4* (105 bp) F: GTTACGGTAAGGAGAGCAGTTT, wordR: CTTTCACTCCAGCCTCCTTC, *BMPR1A* (100 bp) F: GTGCTGTCGGACTGATTTCT, wordR: TGCCATCCAACGAATGCT, *WIF1* (100 bp) F: CAACCTGTTTCAATGGAGGAAC, R: GGCTGATGGCATTTACTGATTT, *OSR2* (140 pb) F: CCACTTCACCAAGTCCTACAA, R: TCTCTTTGGAATGGAT GTACCG. The statistical significance of the data was evaluated through two-tailed Student's *t*-test. *p*-values less than or equal to 0.05 (95% confidence level) were considered as statistically significant differences.

### Western blotting of GLI2/3 proteins

FNPs, MXPs, or MNPs were pooled and lysed in RIPA buffer containing protease inhibitors and phosphatase inhibitors (1 mM Phenylmethylsulfonyl fluoride, 1 mM NaVO_4_, 1X complete protease inhibitor cocktail, EDTA-free), and slightly sonicated with a microprobe to recover chromosome bound GLI2 and GLI3 proteins. BCA assay (Pierce) was used to measure protein concentration of cell lysates. Proteins were boiled with 1X Laemmli sample buffer and run on 6% SDS-PAGE for GLI2 and GLI3, or 12% SDS-PAGE for GAPDH, which later were wet-transferred to Polyvinylidene difluoride (PVDF) membrane. Anti-GLI2, anti-GLI3 (Polyclonal goat IgG 1:500, R&D systems) and anti-GAPDH (FL-335, polyclonal rabbit IgG 1:4000, Santa Cruz Biotechnology) were prepared in 1X TBST (0.1% Tween-20)/6% nonfat milk, as well as secondary antibodies (anti-goat and anti-rabbit, 1:10,000). Proteins were detected by Electrochemiluminescence assay (Amersham ECL Prime, GE Healthcare Life Science).

### DNA binding affinity assay

*PATCHED 1* promoter oligonucleotides were designed according to the location of a Gli binding region (GBR) at −2549 from the TSS site (GGAAGAAGTGTCAGTGTAAGAGTCTCCACGTGGGTGGTCAAGGCCATGGCTGCCTCACGG). 100 pmole of biotin-conjugated positive oligonucleotides and complementary oligonucleotides (Integrated DNA Technologies) were annealed in 1X TE/50 mM NaCl buffer in a PCR cycler and incubated with Dynabeads Streptavidin (M280, Invitrogen). FNPs, MXPs, or MNPs from day 5 control or *ta*^*2*^ embryos were pooled and processed as described for Western blotting. Cell lysates were incubated with oligonucleotides-bound Dynabeads at 4°C for 2 h. Beads were washed with 1 ml RIPA buffer three times and processed for Western Blotting analysis.

### Chromatin immunoprecipitation

FNPs, MXPs, or MNPs from day 5 control or *ta*^*2*^ embryos were harvested, pooled and crosslinked in 1% formaldehyde. Tissue was homogenized in RIPA buffer and sonicated (Bioruptor®; Pico, Diagenode) at 5 cycles of 30 s on/45 s off. Sheared DNA was distributed around 0.3 kb to 1 kb on 1% agarose gel. Cell lysates were pre-cleaned with Dynabeads protein G (ThermoFisher Scientific) and quantified by BCA assay. Dynabeads for immunoprecipitation were blocked with 20 μg/ml Glycogen, 20 μg/ml BSA, 20 μg/ml yeast RNA in RIPA buffer at 4°C for an hour. GLI3 antibody (AF3690, R&D systems) and pre-blocked Dynabeads Protein G were incubated with 90% of cell lysates at 4°C overnight. 10% of cell lysates were kept as Input. Beads were washed with IP wash buffer I (low salt; 50 mM HEPES-KOH pH 7.5, 150 mM NaCl, 1 mM EDTA, 0.1% sodium deoxycholate, 1% Triton X-100), IP wash buffer II (high salt; 50 mM HEPES-KOH pH 7.5, 500 mM NaCl, 1 mM EDTA, 0.1% sodium deoxycholate, 1% Triton X-100), IP Wash Buffer III (LiCl containing buffer; 10 mM Tris-Cl pH 8.0, 250 mM LiCl, 1 mM EDTA, 0.5% Sodium deoxycholate, 0.5% NP-40) and TE buffer (10 mM Tris-HCl pH 8.0, 1 mM EDTA). ChIP samples were reverse-crosslinked by boiling with 10% Chelex-100 (BioRad), and treated with 0.2 mg/ml Proteinase K at 55°C for 30 min. Immunoprecipitated DNA samples were analyzed with quantitative real-time PCR (BioRad) with primers to GBR of target genes. Error bars in all figures represent standard error of the mean (S.E.M.) from five to seven independent experiments. The statistical significance of the data was evaluated through two-tailed Student's *t*-test. *p*-values less than or equal to 0.05 (95% confidence level) were considered as statistically significant differences.

### GBR analysis

Sequences for GBRs from previous publications (Vokes et al., 2007, 2008) were used with a custom perl script to search for all the exact matches of the various possible sequences of the consensus GBRs in the chicken genome. Acquired positions of the motifs in the genome were run through a second custom perl script to search for genes that encompass these motif sites at a distance of 20 kb from either end. Potential GLI targets were confirmed using chromatin-immunoprecipitation (ChIP) assays.

## Results

### The avian ciliopathic mutant, *talpid^*2*^*, has craniofacial anomalies characteristic of a ciliopathy

**Figure 1:**
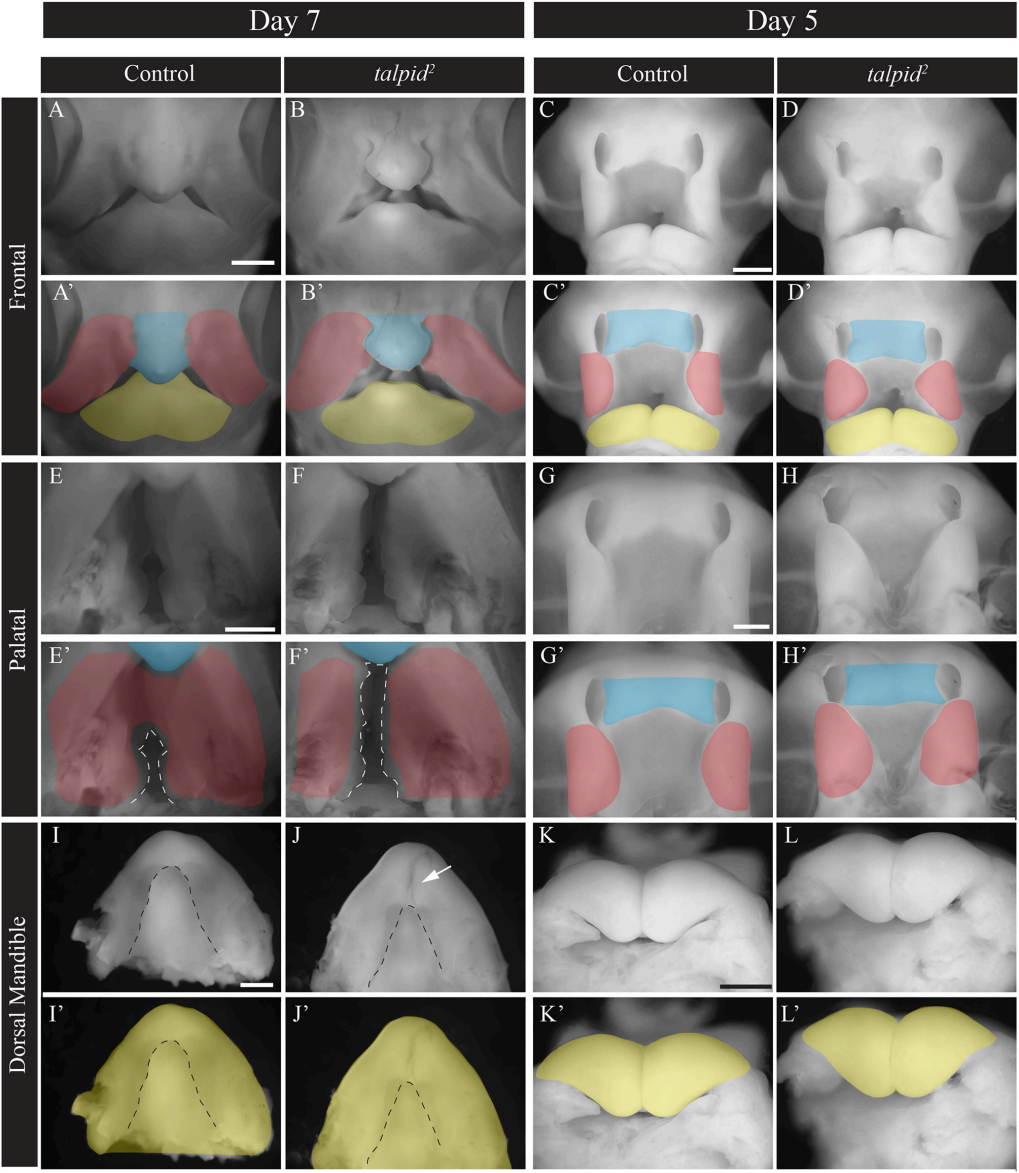
***ta*^*2*^ embryos have craniofacial anomalies common to craniofacial ciliopathies. (A–B')** Frontal view of whole-mount day 7 control and *ta*^*2*^ embryos. **(C,D')** Frontal view of whole-mount day 5 control and *ta*^*2*^ embryos. **(E–F')** Palatal view of day 7 control and *ta*^*2*^ embryo with the forming secondary palate outlined (white dotted line). **(G–H')** Palatal view of day 5 control and *ta*^*2*^ embryo. **(I–J')** Dorsal view of day 7 control and *ta*^*2*^ mandibles with tongue outlined (black dotted line). **(K–L')** Dorsal view of day 5 control and *ta*^*2*^ mandibles. Facial prominences have been pseudocolored as follows: frontonasal prominence (FNP, blue), maxillary prominence (MXP, red), and mandibular prominence (MNP, yellow). Scale bars: 1 mm **(A–J')** and 650 μm **(K–L')**.

To understand the transcriptional networks affected in craniofacial ciliopathies we analyzed the *talpid*^*2*^ (*ta*^*2*^) mutant, a naturally occurring avian ciliopathic mutant that has been established as a model for the human craniofacial ciliopathy, Oral-facial-digital syndrome 14 (Chang et al., 2014; Schock et al., 2015). The *ta*^*2*^ craniofacial phenotype characterized by facial and palatal clefting, micrognathia, and hypoglossia, is fully evident at day 7 (Figure 1). Although our previous work has characterized this phenotype (Chang et al., 2014; Schock et al., 2015), to determine the transcriptional networks that contribute, we first needed to identify when phenotypic onset occurred. At day 7 the frontonasal prominence (FNP) is shorter and wider and frequently does not fuse to adjacent prominences (Figures 1A–B'). Two days earlier at day 5, the MXPs were medially rotated, the nasal pits were larger, thus preventing the proper juxtapositioning of the FNP with adjacent prominences (Figures 1C–D'). Palatal views showed increased patency of the naturally cleft avian secondary palate in *ta*^*2*^ embryos relative to controls at day 7 (Figures 1E–F', dotted white lines). Two days earlier, at day 5, the dysmorphology and malposition of the MXPs is just becoming apparent (Figures 1G–H'). Dorsal views of the developing lower beak showed the MNP of *ta*^*2*^ embryos failed to fuse completely and had a hypoplastic tongue (hypoglossia, dotted black line) (Figures 1I–J', white arrow). At day 5 there was little difference in mandibular growth between control and *ta*^*2*^ MNPs (Figures 1K–L'). From phenotypic evaluations, taken together with the fact that these anomalies were not readily identifiable at day 4, we determined that craniofacial anomalies were initiated at approximately day 5 of development. Thus, to determine the molecular networks responsible for these phenotypes we carried out our analyses in the facial prominences of day 5 embryos.

### Loss of cilia results in aberrant GLI isoform production

**Figure 2:**
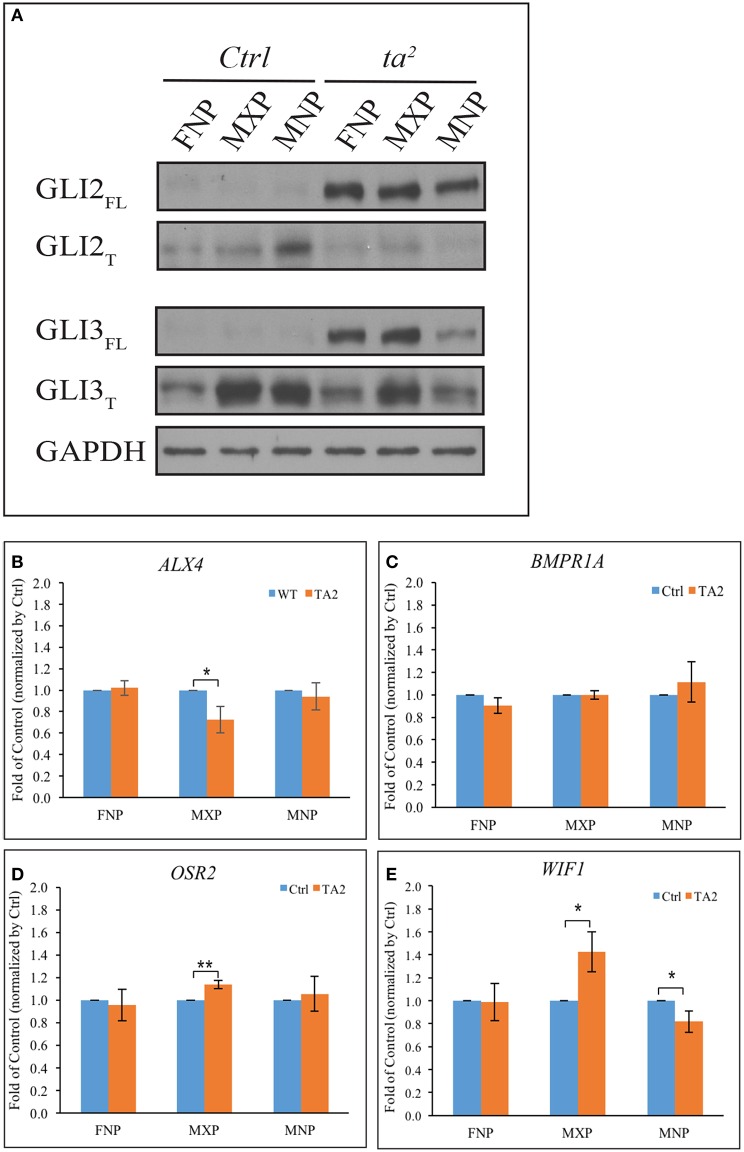
**Excessive GLI_FL_ production in *ta*^*2*^ embryo does not correlate with increased gene activation of GLI targets. (A)** Western blot of GLI2 and GLI3 proteins from the frontonasal prominence (FNP), maxillary prominence (MXP) and mandibular prominence (MNP) of day 5 control and *ta*^*2*^ embryos. GAPDH was used as a loading control. **(B–E)** mRNA-qPCR analyses of *ALX4, BMPR1A, OSR2, WIF1* from FNP, MXP, and MNP of day 5 control and *ta*^*2*^ embryos. The data was normalized by the individual facial prominences of control and *ta*^*2*^ embryos. The asterisks indicate statistically significant differences and are assigned as followed: ^*^P < 0.05, ^**^P < 0.01. Error bars are based on the standard error of the means (S.E.M.). *n* = 4.

Our previous work determined that aberrant ciliogenesis in *ta*^*2*^ embryos disrupted the production of GLI proteins in such a manner that there was increased GLI_FL_ production and decreased GLI_T_ production (Chang et al., 2014). The GLI_FL_ isoform typically goes on to function as an activator, whereas the GLI_T_ isoform goes on the function as a repressor. The excessive production of GLI_FL_ is an extraordinarily common molecular phenotype in ciliopathies, including those with craniofacial phenotypes (Huangfu and Anderson, 2005; Davey et al., 2006; Tran et al., 2008; Tabler et al., 2013). To carefully exam the differences of GLI_FL_ and GLI_T_ protein levels in control and *ta*^*2*^ mutants, we performed Western blot analysis of GLI2 and GLI3 in the three facial prominences affected in the *ta*^*2*^ at day 5. We detected a very low level of GLI2_FL_ and a substantial amount of GLI2_T_ isoforms in the control facial prominences (Figure 2A). The loss of cilia in *ta*^*2*^ embryos disrupted GLI processing and altered the production of GLI2 protein isoforms. In *ta*^*2*^ facial prominences, we detected dramatically increased levels of GLI2_FL_, and low levels of GLI2_T_, relative to control prominences. We next examined the production of GLI3 isoforms. Western blot analyses showed that, similar to GLI2_FL_, GLI3_FL_ was increased in *ta*^*2*^ prominences relative to controls. Contrary to what was observed with the GLI2_T_, GLI3_T_ was readily detectable in both control and *ta*^*2*^ facial prominences. Specifically, *ta*^*2*^ MNP had less GLI3_T_ than control MNP. These data suggested that disrupted ciliogenesis affected the processing of GLI2 and GLI3 in distinct manners, yet the net result was an increase in GLI_FL_ production. We did not observe a change in GLI1 protein levels between control and mutant embryos (Data not shown).

Since the GLI_FL_ isoform typically goes on to act as an activator, these results suggest that craniofacial phenotypes in the *ta*^*2*^ mutant could be caused by increased GLI activator function leading to increased expression of GLI target genes. To test this hypothesis, we utilized an *in silico* approach to look for potential GLI target genes within the avian genome. Using previously published sequences of GBRs (Vokes et al., 2007, 2008) we scanned the chicken genome for possible GLI targets. GBR positions were run through a custom perl script to search for genes that encompass these motif sites at a distance of 100 kb from the 5′ or 3′ end of the gene. From this list we identified several genes known to play a role in craniofacial development (Supplemental Table 1). Confirmation of our *in silico* approach was carried out on selected genes using ChIP assays (Supplemental Figures 1 A–D). Through these analyses, we selected four GLI3 target genes known to be expressed during, and have a role in, craniofacial development: *ALX4* (Beverdam et al., 2001; Mavrogiannis et al., 2001), *BMPR1A* (Li et al., 2011, 2013; Saito et al., 2012), *OSR2* (Lan et al., 2001), and *WIF1* (Hsieh et al., 1999; Darnell et al., 2007). *PTCH1*, a known GLI target, was used as a positive control (Supplemental Figure 1E) and IgG IP as an antibody background control. The data were all normalized to IgG IP percentage, and the genes with relative enrichment >1 were considered positive for GLI3 binding. To determine if increased production of GLI3_FL_ in *ta*^*2*^ facial prominences correlated with increased expression of these target genes, we performed quantitative RT-PCR (qPCR) with mRNA from facial prominences of day 5 control and *ta*^*2*^ embryos (Figures 2B–E). There was not an increase in *ALX4* expression in any of the facial prominences (Figure 2B); however, we detected a significant decrease in *ALX4* expression in the MXP. *BMPR1A* expression was also not significantly increased in any of the developing prominences (Figure 2C). No significant changes in *OSR2* expression were detected in the FNP or MNP; however, a significant increase in expression was observed in the MXP (Figure 2D). Finally, *WIF1* expression was not changed in the FNP, yet was significantly increased in the MXP and significantly decreased in the MNP (Figure 2E). Taken together, these data do not support the idea that increased production of GLI3_FL_ directly and uniformly results in increased expression of GLI targets throughout the facial prominences. Additionally, these data suggest that each facial prominence interprets aberrant GLI production in a unique manner.

### Excess production of GLI_FL_ does not correlate with an increase of GLI_FL_ occupancy at GBRs

**Figure 3:**
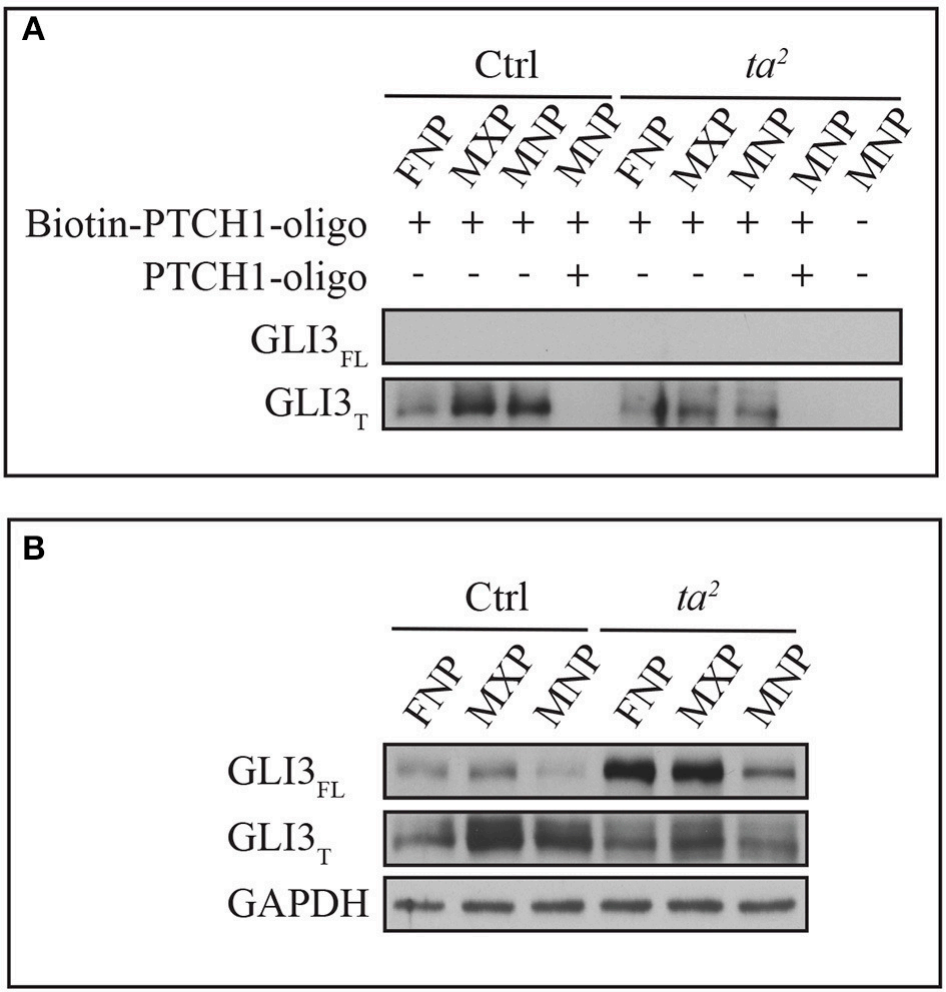
**GLI3_T_ isoform is predominantly recruited to GBR motif of *PTCH1* promoter *in vitro*. (A)** DNA binding affinity assay of GLI3 proteins with frontonasal prominence (FNP), maxillary prominence (MXP), and mandibular prominence (MNP) in day 5 control and *ta*^*2*^ embryos. **(B)** Western blot of GLI3 proteins from the same lysates as **(A)**. GAPDH as loading control of cell lysates.

For GLI_FL_ to function as an activator, it has to occupy the regulatory regions of GLI targets. We wondered if lack of uniform increases of target gene expression in *ta*^*2*^ embryos was due to failure of GLI_FL_ to recognize and occupy GBRs of target genes. To test this hypothesis, we performed an *in vitro* DNA binding assay using the *PATCHED 1* (*PTCH1*) promoter. We synthesized a 60 base pair biotin-labeled oligonucleotide of the *PTCH1* promoter containing an endogenous GLI binding motif found at position −2549 proximally upstream of the transcription start site (TSS). Through high affinity of Biotin-Streptavidin interaction, we were able to evaluate the DNA binding ability of GLI3 isoforms by Western blot. Pre-incubation of non-labeled *PTCH1* oligonucleotides depleted GLI3 protein signals, which confirmed the specificity of the GLI3-GBR interaction. Under the same exposure, the affinity based pull-down assay showed that GLI3_T_ predominantly bound to the biotin-labeled oligonucleotides in both control and *ta*^*2*^ mutants. Interestingly, despite the high level of GLI3_FL_ production in *ta*^*2*^ embryos, GLI3_FL_ failed to bind to the biotin-labeled oligonucleotides (Figures 3A,B). On the contrary, comparison between control and *ta*^*2*^ embryos indicated that the amount of GLI3_T_ binding to the oligonucleotides correlates with the protein concentration (Figures 3A,B). Taken together these data suggest that despite increased GLI3_FL_ production, target gene expression is not increased via increased GLI activator function because GLI3_FL_ does not occupy the GBRs of target genes. In addition, the predominant binding of the GLI3_T_ repressor at GBRs supports the idea of the threshold repression model, in which GLI targets underwent de-repression due to the removal of GLI_T_ repressor.

### GLI3 occupancy at GBRs within the regulatory regions of target genes is altered in *ta^*2*^* mutants

**Figure 4:**
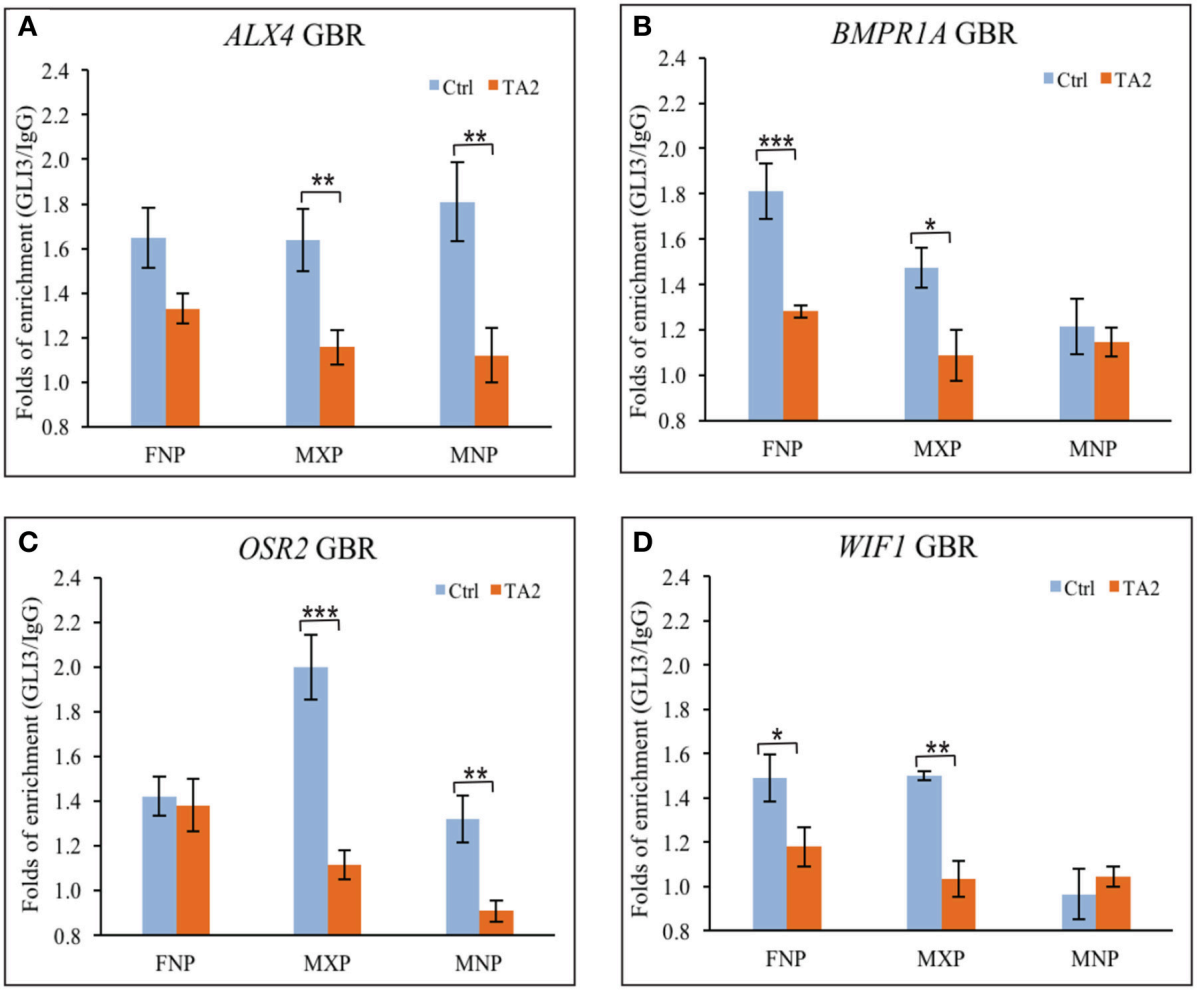
**GLI3 occupancies at the GBRs of GLI targets is reduced in *ta*^*2*^ mutant. (A–D)** ChIP-qPCR analyses of GLI3 precipitated with GLI binding regions of **(A)** *ALX4*, **(B)** *BMPR1A*, **(C)** *OSR2*, and **(D)** *WIF1* in control and *ta*^*2*^ facial prominences. The asterisks indicate statistic significant difference and are labeled as followed: ^*^P < 0.05, ^**^P < 0.01, ^***^P < 0.001. Error bars are based on the standard error of the means (S.E.M.). *n* = 5.

*In vitro* DNA binding affinity assays suggested that there was not an increase of ectopic GLI3_FL_ at GBRs of target genes in *ta*^*2*^ mutants. To determine if GLI3 binding to GBRs was altered *in vivo* in *ta*^*2*^ mutants compared to control embryos, we next performed ChIP-qPCR (Figure 4). In *ta*^*2*^ mutants, GLI3 enrichment at GBRs associated with the craniofacial genes *ALX4* and *OSR2* was reduced in all facial prominences, yet only significantly in the MXP and MNP (Figures 4A,B). GLI3 enrichment at the GBRs associated with *BMPR1A* and *WIF1*, was also overwhelmingly reduced in facial prominences, yet only significantly in the *ta*^*2*^ FNP and MXP (Figures 4C,D). GLI3 binding in the MNP of *WIF1* was below detectable levels in controls, and thus could not be evaluated. These data suggested that GLI3 occupancy at the GBR of target genes is decreased. Our *in vitro* data suggested that GLI3_FL_ did not bind at target gene GBRs (Figure 3). Thus, taken together these data indicate that the observed reduction of GLI3 binding is indicative of reduced GLI3_T_ binding at the regulatory regions of target genes.

Reduced GLI3 binding at GBRs of GLI target genes appeared to be a general trend in *ta*^*2*^ mutants, as several other target genes expressed in the craniofacial complex also exhibited reduced enrichment of GLI3 at associated GBRs (Supplemental Figure 2). Thus, these data suggested that altered expression of GLI targets could be caused by aberrant GLI3_T_ binding in *ta*^*2*^ mutants. Interestingly, there was not always a direct correlation with loss of GLI3_T_ repressor binding and increased gene expression. Furthermore, different results among the three facial prominences examined pointed to context—and tissue-specific regulation of GLI targets (Supplemental Figures 1A–D). Several studies have proposed the possibility that GLI proteins work together with co-regulators to influence expression of targets genes (Brewster et al., 1998; Koyabu et al., 2001; Mizugishi et al., 2001; Lee et al., 2010; Peterson et al., 2012). To determine if target gene expression in the craniofacial complex was influenced by the action of GLI co-regulators, we next examined the proximity of GBRs to motifs for potential GLI co-regulators.

### Motif analyses identified sequences for known GLI co-regulators frequently co-localize with GBRs

**Figure 5:**
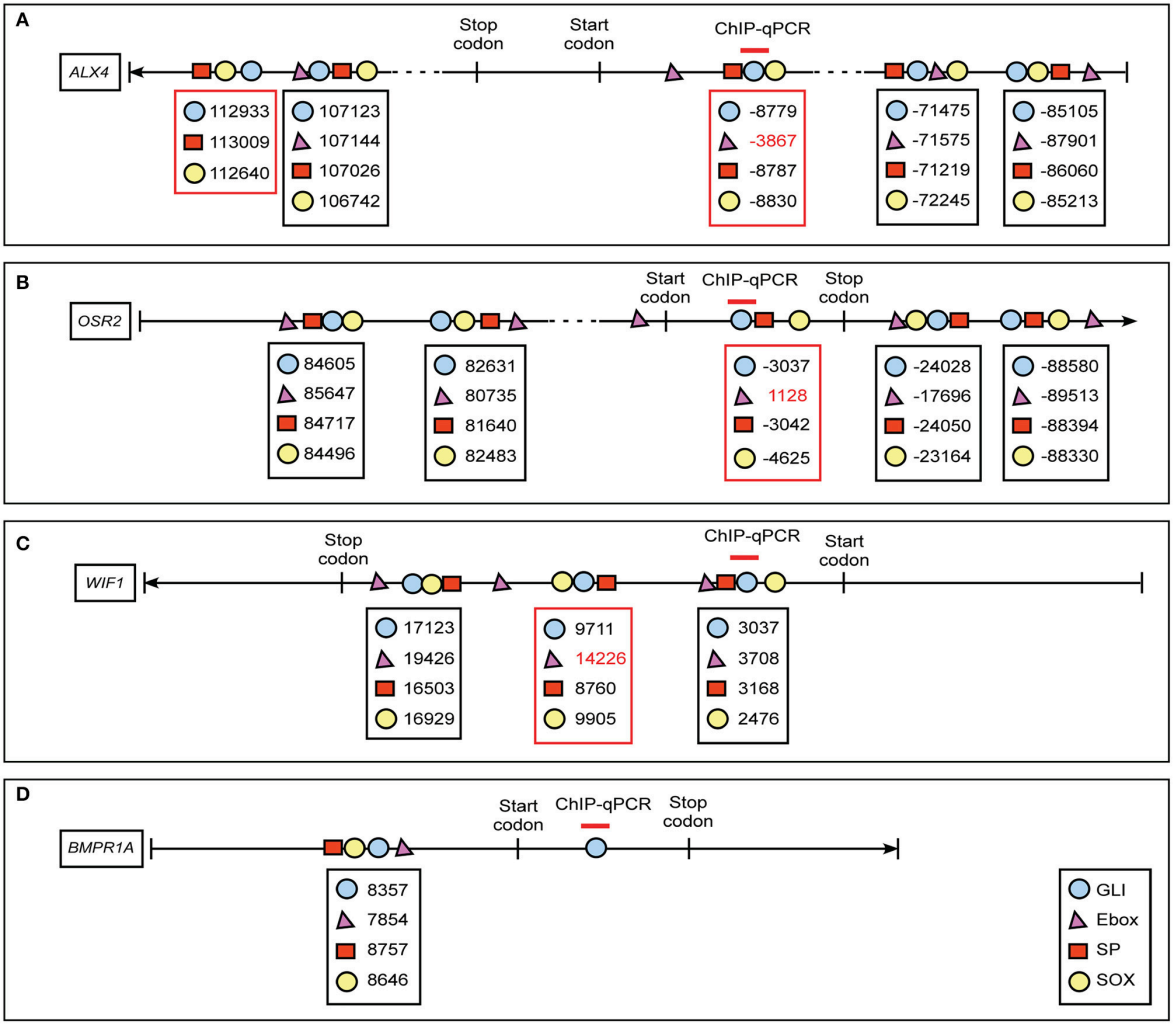
**Schematic diagram of co-regulator motif clusters near GBRs of *ALX4*, *OSR2*, *WIF1* and *BMPR1A***. *In silico* analyses predict location of GLI (blue circle), E-box (magenta triangle), SP (red rectangle), and SOX (yellow circle) sites in GLI target genes **(A)** *ALX4*, **(B)** *OSR2*, **(C)** *WIF1*, **(D)** *BMPR1A*. We defined 5′-untranslated region (UTR), gene and 3′-UTR as intragenic region, < 1 kb upstream of transcription start site (TSS) is promoter region, 1–20 kb away from TSS as proximal regulatory region, and < 100 kb away from TSS as distal regulatory region. The numbers labeled below the symbols indicate the positions of the motifs according to the distance away from TSS sites. The position upstream of TSS site is assigned a negative symbol. The area amplified by primer sets used for ChIP-quantitative PCRs are labeled above the specific GLI binding motif (red line). Clusters of four motifs are highlighted in a black box, clusters of three motifs are highlighted in a red box.

GBRs have previously been identified in close proximity to binding motifs for other transcription factors, including members of the bHLH, SP, and Sox families (Vokes et al., 2008; Peterson et al., 2012; Aberger and Ruiz I Altaba, 2014). Furthermore, the co-occupancy and cooperativity of GLI with SOX transcription factors was previously shown to be essential for activating neural gene expression signatures (Peterson et al., 2012). To determine if expression of our identified GLI targets could be influenced by the presence or absence of GLI co-regulators, we first examined the genomic sequence around the GBRs of our target genes for E-box, SP, and SOX binding motifs. (Figure 5, Supplemental Figure 3). We defined the area < 1 kb upstream of the transcription start site (TSS) as the promoter region, < 20 kb away from the TSS as proximal upstream or downstream, and < 100 kb away from the TSS as distal upstream or downstream. We analyzed the sequence surrounding GBRs in our four selected GLI target genes for sequences predictive of E-box, SP, and SOX binding. All four of our identified GLI target genes contained at least one motif cluster containing a GBR, E-box, SP, and SOX binding site within 1 kb of each other (Figures 5A–D). Several other clusters containing three of the four motifs were also identified (Figures 5A–C; red box). The close proximity of these binding motifs suggested that expression of GLI targets in the developing craniofacial complex could be influenced by the cooperative function of GLI isoforms and co-regulator proteins.

### GLI co-regulators have a prominence specific expression pattern that changes when primary cilia are lost

**Figure 6:**
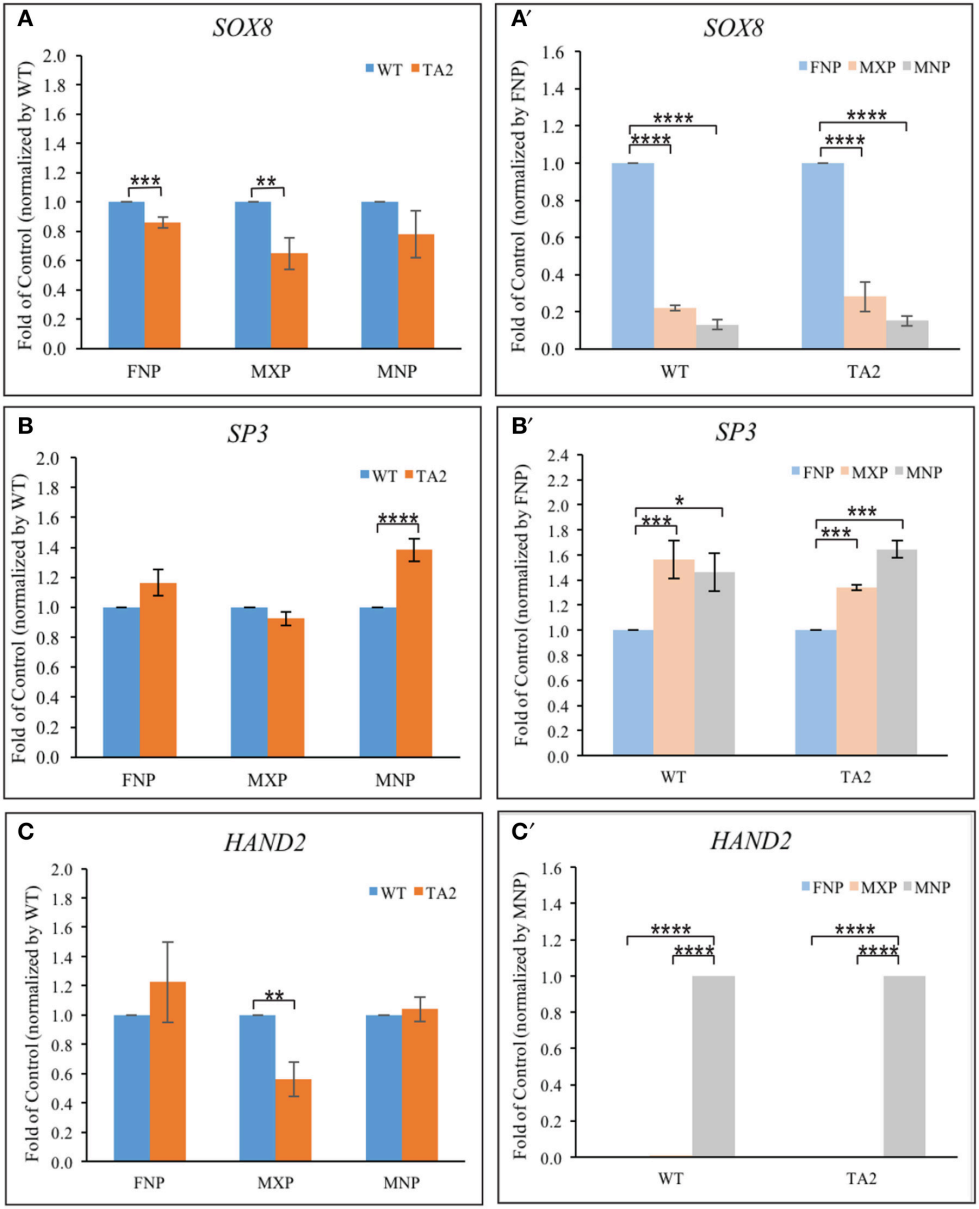
**Prominence-specific expression of *SOX8*, *SP3*, and *Hand2***. Quantitative RT-PCR analyses of transcription factors (*SOX8, SP3* and *Hand2*) based on the comparison between control and *ta*^*2*^ embryos **(A–C)** or individual facial prominences **(A'–C')**. The asterisks indicate statistical significance and are labeled as followed: ^*^P < 0.05, ^**^P < 0.01, ^***^P < 0.001, ^****^P < 0.0001. Error bars are based on the standard error of the means (S.E.M.). *n* = 4.

Several transcription factors synergistically cooperate with GLI proteins to influence GLI target gene expression (Aberger and Ruiz I Altaba, 2014). Their co-occupancy at the promoter of GLI targets is required for the optimal activation/repressor. ChIP-based, high-throughput analyses uncovered several transcription factor motifs located close to GBRs in the cis-regulatory modules of GLI targets. Specifically, binding sequences for Sox (Peterson et al., 2012), bHLH (Lee et al., 2010), and SP proteins (Vokes et al., 2008) have been shown to exist in very close proximity to GBRs. Our *in silico* analyses confirmed these sequences exist in near GBRs in four previously identified GLI-targets. We hypothesized that differential expression of these co-regulators could contribute to the differential gene expression of GLI targets in both control and *ta*^*2*^ mutant embryos. We first investigated the expression of genes that could bind to motifs found in close proximity to GBRs, specifically *SOX8, SP3*, and *HAND2* (Figures 6A–C). *SOX8* expression was significantly reduced in the FNP and MXP of *ta*^*2*^ mutants, yet was not significantly altered in the MNP (Figure 6A). *SP3* expression was significantly increased in *ta*^*2*^ MNP, yet not changed in the FNP and MXP (Figure 6B). *HAND2* expression was reduced in the MXP, but not significantly changed between control and *ta*^*2*^ FNPs and MNPs (Figure 6C). Thus, differential expression of GLI co-regulators could possibly contribute to altered target gene expression in *ta*^*2*^ mutants and explain why changes in GLI_FL_ and GLI_T_ isoforms do not uniformly or directly correlate with changes in target gene expression.

Our previous analyses indicate that GLI target gene expression changed in a prominence specific manner (Chang et al., 2014). We wondered if differential expression of potential GLI co-regulators could contribute to this prominence-specific expression changes. *SOX8* was robustly expressed within the FNP, with levels significantly higher than those in the MXP or MNP (Figure 6A'). *SP3* was more robustly expressed in the MXP and MNP, relative to the FNP (Figure 6B'). *HAND2* was robustly and exclusively expressed in the MNP (Figure 6C'). Thus, each of these potential co-regulators has a prominences specific expression pattern that could differentially influence GLI target gene expression. Collectively, the proximity of binding motifs, coupled with the differential expression of these co-regulators could possibly contribute to altered expression of GLI targets in *ta*^*2*^ embryos.

## Discussion

Craniofacial ciliopathies are a rapidly growing group of disorders that severely impact craniofacial growth and development. Currently, there are little to no therapeutic options for these conditions. Although the molecular mechanism behind these disorders remains nebulous, many ciliopathies have aberrant production of GLI_FL_ and GLI_T_ isoforms. (Huangfu and Anderson, 2005; Davey et al., 2006; Tran et al., 2008; Tabler et al., 2013; Chang et al., 2014). Herein, we attempted to identify the mechanism by which aberrant GLI protein production impacts craniofacial development. To do so we used the avian *ta*^*2*^ model, which has recently been characterized as a bona fide model for the human craniofacial ciliopathy Orofacial-digital syndrome 14 (Schock et al., 2015). *In silico* and ChIP assays identified GLI target genes in the avian genome that play a role in craniofacial development. RT-qPCR of mRNA levels verified some significant changes in the expression of these GLI targets in the developing facial prominences, yet there was not a clear, linear relationship between changes in GLI isoform production and target gene expression. Motif cluster analysis supported the hypothesis that GLI proteins work in concert with co-regulators. GBRs associated with GLI target genes were found to be situated within 1000 bp of binding motifs for several, previously identified GLI co-regulators. Differential expression within developing facial prominences of predicted co-regulators supported a hypothesis in which expression of GLI target genes is dependent upon the cooperative function of GLI isoforms and co-regulator proteins. Together, these data provide a better understanding of the complex nature of GLI-mediated transcription that occurs in normal and ciliopathic craniofacial development.

### GLI binding regions are present throughout the avian genome in genes that affect craniofacial development

Work in other species has identified GBRs and examined the role of GLI proteins as transcription factors that affect gene expression in numerous signaling pathways (Vokes et al., 2007, 2008). Three models currently exist to explain GLI_FL_/GLI_T_ function (Falkenstein and Vokes, 2014). First the GLI_FL_::GLI_T_ ratio sensing model suggest that the relative levels of GLI_FL_ to GLI_T_ is integrated and results in graded levels of transcription of targets. This model suggests that response to changes in ratio, rather than concentration, affect target gene expression. The threshold activation model suggests that, rather than ratio of GLI_FL_::GLI_T_, a threshold-specific concentration of GLI_FL_ activates target gene expression (Oosterveen et al., 2012). The threshold repression model depends on the removal of the GLI_T_ repressor from target genes, allowing for transcriptional activation by other transcription factors to initiate gene expression. From our results, mRNA expression of most target genes was not dramatically altered despite excessive GLI_FL_ production in *ta*^*2*^ mutant, indicating that the ratio sensing model cannot explain our observation in *ta*^*2*^ mutant. Secondly, the accumulated GLI3_FL_ in the nucleus was expected to bind and activate target gene in the threshold activation model; however, instead we found reduced occupancy of GLI3 at target genes, along with lack of detectable GLI_FL_ binding to GBRs (Figures 3, 4), suggesting that a dysfunctional GLI_FL_ that fails to promote GLI_A_-mediated activation is produced under ciliopathic conditions. Thus, these data do not support the threshold activation model for GLI_FL_ function. Conversely, our results showed that reduced GLI_T_ production could lead to a de-repression of target genes and potentially a gene activation, when the required transcription factors are available. The threshold repression model seems to be more applicable to the observation owing to less GLI3 occupancy and the failure of GLI_FL_ to GLI_T_ conversion. A more in depth understanding of GLI mechanisms of action will require tools with the ability to definitively decipher between GLI_FL_ and GLI_T_ *in vivo*.

### Excessive GLI_FL_ production does not equate to increased GLI_A_ activity in ciliary mutants

A number of disorders identified as ciliopathies have craniofacial abnormalities including Oral-facial-digital syndrome, Joubert syndrome, Bardet-Biedl syndrome, Meckel-Gruber syndrome, Ellis-van Creveld syndrome (Zaghloul and Brugmann, 2011). The phenotypes for syndromes such as these, while not identical, do have several phenotypes indicative of aberrant SHH signaling including widening of the midface, cleft lip/palate, micrognathia, craniosynostosis and oral/dental anomalies (Zaghloul and Brugmann, 2011). Increased production of GLI_FL_ isoforms has been observed in several ciliary mutants (Huangfu and Anderson, 2005; Liu et al., 2005; Humke et al., 2010), thus a common interpretation of these data is that ciliopathic phenotypes were due to either increased activator function or skewed GLI_FL_::GLI_T_ ratio in favor of GLI_FL_. Despite these common interpretation regarding the molecular mechanism causing ciliopathic phenotypes, examination of subsequent levels of GLI processing, binding, and transcriptional function in ciliary mutants has not been performed.

Our studies are among the first to examine how and if GLI proteins function in the developing craniofacial complex of ciliary mutants; however, some questions remain regarding the mechanistic reasons as to why GLI_FL_ appears not to function in ciliary mutants. Prior to processing, GLI proteins associate with Suppressor of Fused (SUFU), a conserved protein known to regulate the activity of GLI proteins via modulating GLI processing, stabilization and subcellular localization (Barnfield et al., 2005; Humke et al., 2010; Tukachinsky et al., 2010; Wang et al., 2010). In the presence of a SHH signal, the SUFU-GLI_FL_ complex traffics through the cilium (Eggenschwiler and Anderson, 2007). Activated ciliary SMO then works through KIF7 to promote the dissociation of the inhibitory SUFU-GLI_FL_ complex (Humke et al., 2010; Tukachinsky et al., 2010; Li et al., 2012). Free GLI_FL_ is then processed into an activator and moves to the nucleus to activate downstream targets. In the absence of the SHH ligand, SMO is not translocated into the cilium and thus cannot antagonize SUFU. SUFU remains in complex with GLI_FL_, GLI_FL_ is proteolytically processed into GLI_T_ and the SUFU-GLI_T_ complex moves to the nucleus where it recruits the Sap18-Sin3 co-repressor complex to repress GLI target genes (Ding et al., 1999; Kogerman et al., 1999; Cheng and Bishop, 2002; Paces-Fessy et al., 2004). Furthermore, our recent work with the murine ciliary mutant, *Kif3*^*fl*/*fl*^;*Wnt1-Cre* shows increased SUFU production and nuclear localization, as well as enhanced association of SUFU with GLI3 (Chang et al., in press). We hypothesize a similar mechanism is at play in *ta*^*2*^ mutants. Specifically, excessive GLI_FL_ produced in *ta*^*2*^ mutants cannot dissociate from SUFU because the complex cannot undergo ciliary trafficking. We further hypothesize that lack of dissociation prevents GLI_FL_ from occupying GBRs and directly activating target gene transcription. Our future studies will address the association of GLI_FL_ and SUFU in *ta*^*2*^ mutants and determine if their maintained association contributes to ciliopathic phenotypes.

### GLI-binding to target genes occurs in a prominence specific manner

Frequently the craniofacial complex is thought of as a singular organ system; however, the prominences that make up the face develop independently prior to their fusion. There is evidence to support that these prominences have distinct molecular profiles and develop as separate developmental fields (Brugmann et al., 2007). Our ChIP-qPCR results detected differential GLI binding to target genes in facial prominences of control embryos (Figures 2B–D). Further, the expression of co-regulators also followed a prominence specific pattern in both control and *ta*^*2*^ embryos (Figures 6A'–C'). These data supported the hypothesis that during normal development, as well as when cilia are lost, each facial prominence uses a unique mechanism to transduce a SHH-dependent GLI signal. Based on our examination of co-regulators, we hypothesize that there is a combinatorial code of GLI isoforms and co-regulators that work together to precisely regulate target gene expression. Thus, when cilia are lost and GLI production is altered, target gene expression is dictated by how the combinatorial code of remaining GLI isoforms and co-regulators function together.

In sum, our data suggest that the increased or decreased production of GLI isoforms alone is not sufficient to explain how target gene expression will be altered. To understand the molecular mechanisms responsible for ciliopathic, GLI-mediated phenotypes, future studies will have to account for tissue specificity, the presence or absence of co-regulators and the mode of GLI function (activation or de-repression) to begin to address this process.

## Author contributions

YC designed and tested all primers, and performed and interpreted all ChIP and qPCR experiments, PC performed *in silico* GBR analysis, ES harvested and photographed embryos, SB conceived the project, analyzed data and wrote the manuscript with input from all authors.

## Funding

This work was funded by the National Institutes of Health, National Institute of Dental and Craniofacial Research (NIDCR) [R00-DE01985 and [R01-DE023804] to SB] and by C.T.O. and Research in Progress (RIP) funds from the Cincinnati Children's Research Foundation to SB.

### Conflict of interest statement

The authors declare that the research was conducted in the absence of any commercial or financial relationships that could be construed as a potential conflict of interest.

## Supplementary material

Supplementary Figure 1 **GLI3 binding to GLI targets is confirmed by ChIP**. ChIP-qPCR analyses of GLI3 precipitated with GLI binding regions of *ALX4* **(A)**, *BMPR1A* **(B)**, *OSR2* **(C)**, *WIF1* **(D)**, and *PTCH1* **(E)** in facial prominences of control embryo. Error bars are based on the standard error of the means (S.E.M.). *n* = 3.

Supplementary Figure 2 **ChIP-qPCR analyses of GLI3 precipitated with GLI binding regions of *PTCH1***(A)**, *TFAP2A***(B)**, *CTNNB1***(C)**, *WNT2B***(D)**, *DKK1***(E)**, *FGFR***(F)**, *BMP2***(G)**, *BMP4***(H)**, *BMP7***(I)**, and *EPHA4***(J)** in control and *ta*^*2*^ facial prominences**. Error bars are based on the standard error of the means (S.E.M.). *n* = 3.

Supplementary Figure 3 **The schematic of the clusters of transcription factor motifs in GBRs of GLI targets**. The *in silico* analyses of GLI (blue circle), E-box (magenta triangle), SP site (red rectangle), and SOX site (yellow circle) in the GBRs of GLI targets. We defined 5′-untranslated region (UTR), gene and 3′-UTR as intragenic region, < 1 kb upstream of transcription start site (TSS) is promoter region, 1–20 kb away from TSS as proximal regulatory region, and < 100 kb away from TSS as distal regulatory region. The numbers labeled below the symbols indicate the positions of the motifs according to the distance away from TSS sites. The position at the upstream of TSS site is assigned a negative symbol. The primer sets used for ChIP-quantitative PCRs are labeled above the specific GLI binding motif (red line). Clusters of four motifs are highlighted in a black box, clusters of three motifs are highlighted in a red box.

Supplementary Table 1 **Identified Gli target genes**.
