# Supplementary figures and images for: Understanding Mechanisms of GLI-Mediated Transcription during Craniofacial Development and Disease Using the Ciliopathic Mutant, talpid2
Source: Front Physiol. 2016 Oct 17;7:468. doi: 10.3389/fphys.2016.00468 (PMC5065992; doi:10.3389/fphys.2016.00468)

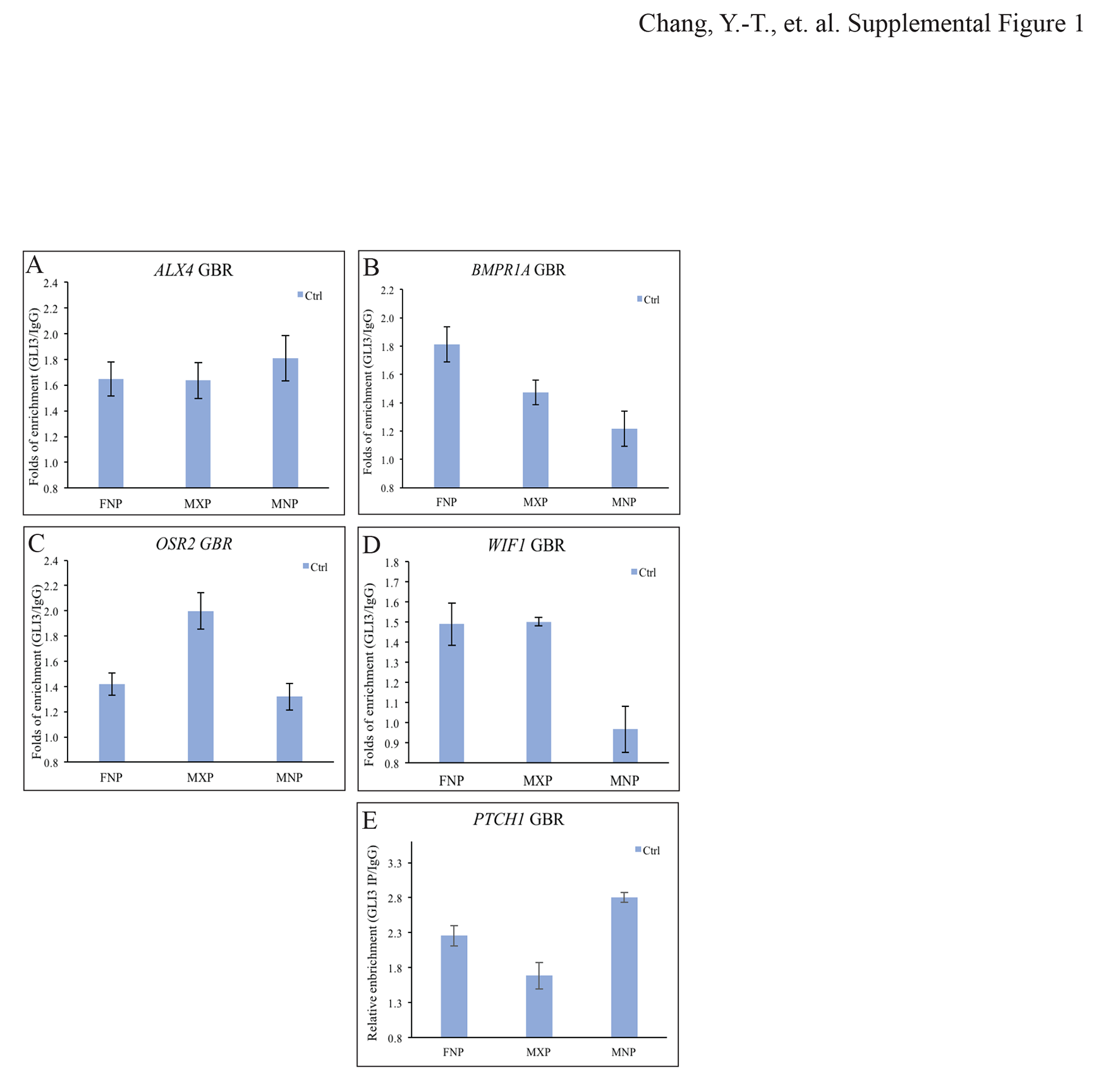

Supplement: Supplementary Figure 1 — GLI3 binding to GLI targets is confirmed by ChIP. ChIP-qPCR analyses of GLI3 precipitated with GLI binding regions of ALX4 (A), BMPR1A (B), OSR2 (C), WIF1 (D), and PTCH1 (E) in facial prominences of control embryo. Error bars are based on the standard error of the means (S.E.M.). n = 3. [file Image1.TIF]

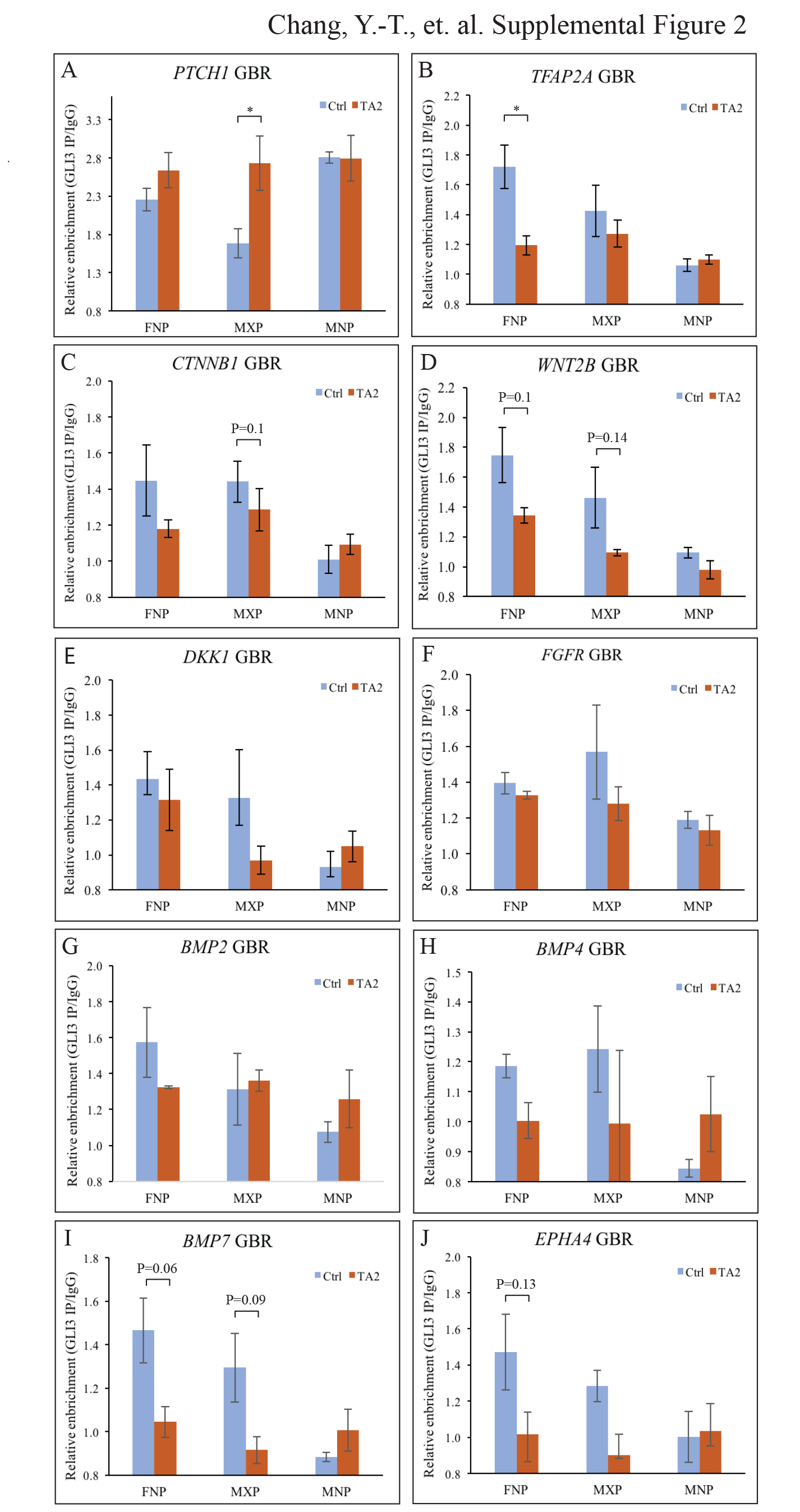

Supplement: Supplementary Figure 2 — ChIP-qPCR analyses of GLI3 precipitated with GLI binding regions of PTCH1(A), TFAP2A(B), CTNNB1(C), WNT2B(D), DKK1(E), FGFR(F), BMP2(G), BMP4(H), BMP7(I), and EPHA4(J) in control and ta2 facial prominences. Error bars are based on the standard error of the means (S.E.M.). n = 3. [file Image2.TIF]

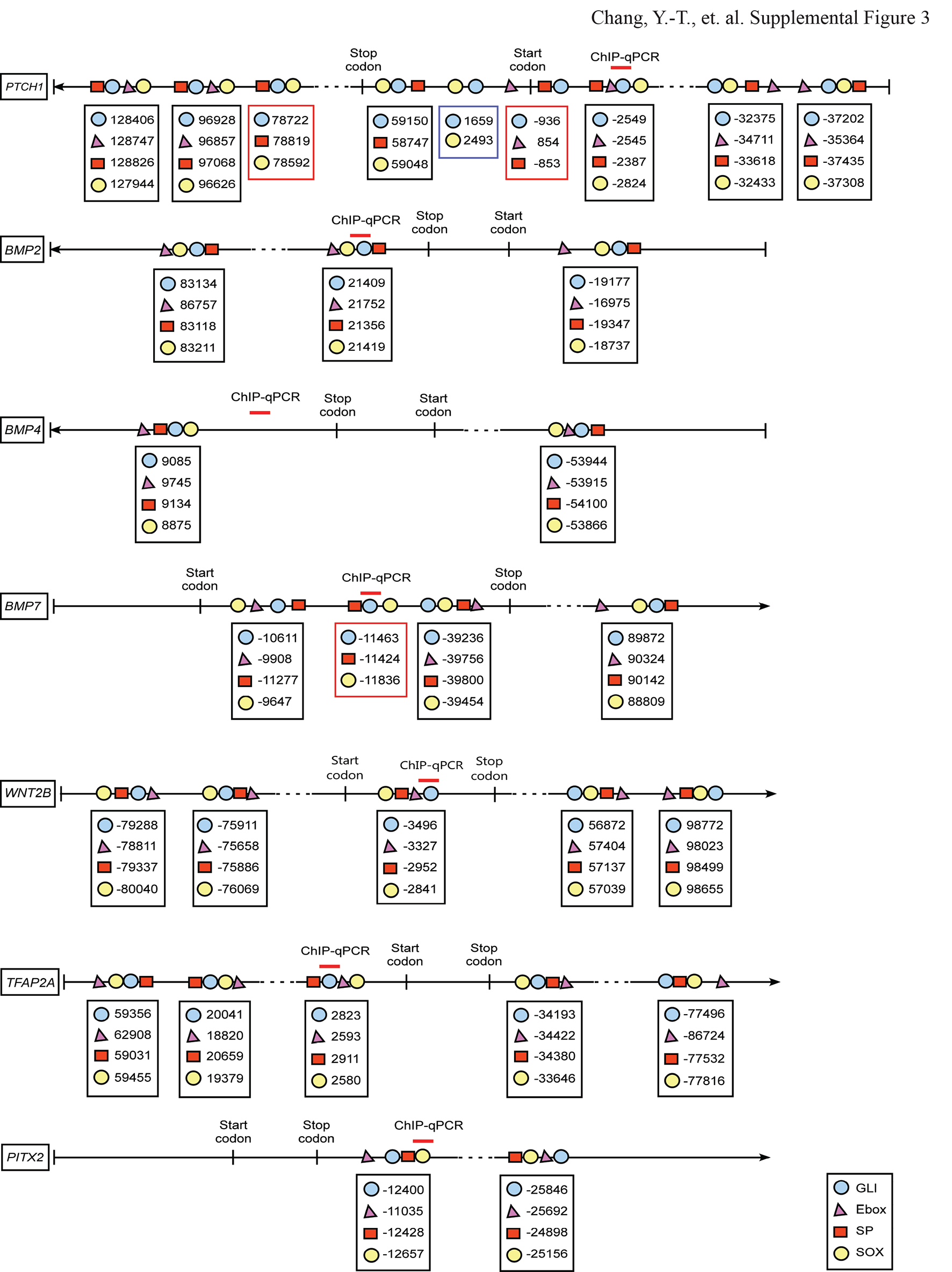

Supplement: Supplementary Figure 3 — The schematic of the clusters of transcription factor motifs in GBRs of GLI targets. The in silico analyses of GLI (blue circle), E-box (magenta triangle), SP site (red rectangle), and SOX site (yellow circle) in the GBRs of GLI targets. We defined 5′-untranslated region (UTR), gene and 3′-UTR as intragenic region, < 1 kb upstream of transcription start site (TSS) is promoter region, 1–20 kb away from TSS as proximal regulatory region, and < 100 kb away from TSS as distal regulatory region. The numbers labeled below the symbols indicate the positions of the motifs according to the distance away from TSS sites. The position at the upstream of TSS site is assigned a negative symbol. The primer sets used for ChIP-quantitative PCRs are labeled above the specific GLI binding motif (red line). Clusters of four motifs are highlighted in a black box, clusters of three motifs are highlighted in a red box. [file Image3.TIF]
